# Open to the public: paywalls and the public rationale for open access medical research publishing

**DOI:** 10.1186/s40900-020-0182-y

**Published:** 2020-02-28

**Authors:** Suzanne Day, Stuart Rennie, Danyang Luo, Joseph D. Tucker

**Affiliations:** 1 Institute for Global Health and Infectious Diseases, 130 Mason Farm Road, University of North Carolina at Chapel Hill, Chapel Hill, 27599 USA; 2 Department of Social Medicine, 333 South Columbia Street, University of North Carolina at Chapel Hill, Chapel Hill, 27516 USA; 3 Center for Bioethics, 333 South Columbia Street, University of North Carolina at Chapel Hill, Chapel Hill, 27516 USA; 4 Zhitong Guangzhou LGBT Center, Guangdong Provincial Dermatology Hospital, Lujing Road, Luhu Park, Yuexiu, Guangzhou, Guangdong China; 5 School of Medicine, 321 South Columbia Street, University of North Carolina at Chapel Hill, Chapel Hill, 27516 USA; 6 Faculty of Infectious Diseases, Keppel Street, London School of Hygiene and Tropical Medicine, London, WC1E 7HT UK

**Keywords:** Open access, Public stakeholders, Accountability, Research ethics

## Abstract

Public voices have largely been absent from the discussions about open access publishing in medical research. Yet the public have a strong interest in ensuring open access of medical research findings because of their roles as funders, advocates, research participants, and patients. By limiting access to research outputs, the current publishing system makes it more difficult for research to be held accountable to the public. Paywalls undermine the work of public advocacy, which requires open access in order to lobby for policy changes and research funding. Research participants generously give their time and energy to research studies with the assumption that the results will be broadly disseminated. Finally, members of the public have a stake in open access publishing as a resource for health information and decision-making. This commentary explores these crucial roles of the public in order to develop a public rationale for open access medical research. We outline a critique of the current academic publishing ecosystem, re-focus the open access debate from a public perspective, and respond to some of the arguments against public open access. Although open access to medical research is not a panacea, removing paywalls and other barriers to public access is essential. The public are critical stakeholders of medical research data.

## Plain English summary

Open access is a publishing model that makes research findings freely available to everyone. However, a large portion of medical research continues to be published behind paywalls that limit access to research findings. Having open access to medical research is important not only for research scientists, but also for members of the general public. Medical research is often made possible through public funds, therefore members of the public should be able to access the research results. Open access is also important in order for the public to have enough information to advocate for changes to policies and research funding. Open access can help to broadly distribute research findings that were made possible through the time and efforts of research study participants. Finally, open access to medical research can help members of the public to have more information for making decisions about their health. In this paper, we outline the problems with publishing medical research behind paywalls, explore the importance of open access to the public, and respond to some of the arguments against public open access. We make practical suggestions to expand open access of medical research.

## Introduction

At an annual meeting for a public-funded medical research project, attendees who had been invited to serve as patient partners on the project expressed with great emotion the urgent need for better communication between academic researchers and community members. For these patients, it was much more than just a study. This was an opportunity to be meaningfully involved in the research process and to correct historical practices of exploitation and communication failures because many of the patient partners came from marginalized indigenous, rural and remote communities. In response to this experience at the annual meeting, our research team made a promise to publish all of our research results only in open access journals to facilitate public dissemination and sharing. We kept this promise to address our patient partners’ concerns. Knowing that the patient partners we heard at the meeting represented a wider public with whom academic research has so often failed to communicate, how could we publish behind a paywall? Open access was a small but crucial step towards honoring the voices of the patient partners who were in the room that day.

Despite the growing conflict between for-profit publishers and public institutions [1], public voices are largely absent from this discussion. Yet members of the public have a strong interest in advancing open access. In making the results of research freely accessible to the broader public, open access publication enhances transparency and public knowledge, and thus is crucial for fostering patient and public engagement with academic endeavors. Additionally, open access publishing clearly has important implications for public health. Open access allows medical evidence that can impact the policies and practices that shape population health to be widely distributed and freely available to all – including not only academic researchers, but also medical professionals, policy makers, and laypersons [2]. Yet besides acknowledging the broader public health benefits of open access, the ways that lay members of the public may use and benefit from open access medical research are rarely examined. Arguments in favor of open access have tended to instead focus on peer-to-peer information sharing between academics, downplaying the importance of public access to medical research findings as being of secondary benefit [3].

To date, few have considered the need for open access publishing beyond academia [4–6]. One notable effort to advance open access to research for the sake of public benefit has been the *Report of the Working Group on Expanding Access to Published Research Findings* (the Finch Report), submitted to the UK government in 2012. In this report, the Finch group explicitly acknowledged the goal of expanding open access to research as a benefit for the public. These benefits are not merely abstract improvements in public knowledge, for the limited existing research suggests that non-academics both prefer and make use of open access medical research when it is made available. For example, a study of Dutch laypersons found that participants had an interest in medical treatment research being freely available for citizens to access at will, particularly when faced with a medical problem [7]. Additionally, a study of UK medical charities found that staff laypersons extensively made use of open access medical research to assist with tasks such as managing research grants and communicating research findings [8].

This evidence highlights the importance of grounding arguments in favor of open access to medical research beyond peer-to-peer sharing, as it is members of the general public who represent the largest group of stakeholders in the open access debate. The public has a strong interest in ensuring open access of medical research findings because of their roles as funders, advocates, research participants, and patients. In this paper, we examine the current system of journal paywalls and describe why the public should be engaged in discussions on open access medical publishing.

### Paywalls and the current open access science environment

Paywalls remain common in medical research. A review estimated that only 28% of all scholarly publications are currently open access [9], meaning that the vast majority of academic knowledge remains inaccessible without a paid individual or institutional subscription. While just over half of all biomedical research was found to be open access, substantial variations were found by discipline; for example, 84% of publications in tropical medicine were open access, while only 7% of pharmacy publications were open access [9]. Another study focused on global health research found that 42% of scholarly articles were published behind a paywall [10]. Paywalls thus continue to represent a substantial barrier to freely access medical knowledge.

Lingering resistance to open access medical research is likely related to the fact that academic publishing is a highly profitable business. Annual revenues from English-language science, technology and medical publishing journals in 2017 were estimated to be USD$10 billion [11]. This profit is primarily concentrated in the hands of a small number of publishers; in 2013, 53% of all natural and medical science publications were published by the five largest for-profit academic publishing companies [12]. By cornering the supply of a relatively unique product, the top commercial publishers are able to charge increasingly high annual fees for access to publications, leaving subscribers with little ability to negotiate [12].

The most obvious stakeholders for whom paywalls pose a problem are research scientists, universities, and libraries. In the absence of universal open access, the ability to obtain information on the latest advancements in one’s field for research or teaching purposes depends on the ability of one’s institution to pay for a sufficient range of increasingly expensive journal subscriptions. The burden of increasing publishing costs is being felt across even large, well-funded institutions. For example, the library of Harvard University announced that the estimated $3.5 million in annual subscription costs were financially unsustainable and encouraged publishing in open access journals as a way to push back [13]. Some institutions have also attempted to resist rising publishing costs by unbundling big deal subscription contracts to save on fees, or cancelling subscriptions altogether (SPARC, [14]). For example, after months of counter proposals in negotiating the terms of contract renewal, The University of California ultimately cancelled its subscription to Elsevier in March 2019 when the publishing giant refused to reconsider terms that would result in higher costs to the university while simultaneously reducing access, excluding content, and limiting financial support to authors [15].

Paywalls not only impact the ability of researchers to access information, they also subsequently reduce the ability of researchers to have their work widely viewed. In a study comparing article usage data, paywalled articles received fewer page views, fewer citations, and less social media attention compared to open access articles [16]. Additionally, paywalls exacerbate the already substantial inequalities in scholarly resources between the global north and global south [17], and raise challenging ethical questions about a for-profit approach to knowledge acquisition [18]. But amidst these challenges, it is important to recognize that the general public is also a stakeholder in the struggle for expanding open access to academic publications – particularly in medical research.

### Public funders, public accountability

The public directly (e.g., crowdfunding) and indirectly (e.g., taxes to governments) funds a substantial portion of medical research. This financial support brings with it a level of obligation to disseminate research results back to the paying public [19]. Many government funders now require open access publishing as a condition of holding grants. The United States, Canada, the United Kingdom, and the European Commission all have various levels of requirements for open access outputs (ROARMAP, [20]), and Plan S by cOAlition S will extend this commitment in other countries [21]. Underlying these policies is a recognition of the obligation to report research findings to the public. The public funding argument is one of the most visible ways that public advocates have organized and lobbied for expanding open access to scientific research, resulting in such efforts as the Taxpayer Alliance for Access [22] and the Access2Research petition [23].

However, sharing research results with the public is not only a matter of knowledge dissemination back to those who helped to pay for it. It is also one of accountability. While researchers are required to provide detailed progress reports to funding bodies, open access publishing presents one of the few opportunities for the public to understand the impact that public money has had on advancing medical research. By limiting access to research outputs, paywalls it more difficult for research to fulfill its obligations and to be held accountable by those whose funding has made it possible.

### Access to medical research findings and public advocacy

Paywalls also undermine public advocacy and involvement in medical research. Advocacy requires open access in order for members of the public to lobby for policy changes and research funding, as well as to identify potential research harms and make calls for greater inclusion of public perspectives. This concern applies not only to publicly-funded medical research, but also privately-funded medical industry research, such as the results of drug trials funded by pharmaceutical companies and studies assessing new medical technologies. While community stakeholder engagement strategies may go some way towards ensuring that public advocates’ voices are considered in the design and conduct of medical research [24], the ability to respond to the research is limited to the extent that the results – whether positive or negative – are hidden from public view. So too is the investigative and reporting work of health journalists, who keep the public informed about the latest medical research advances. From the perspective of public advocacy, the need for open access to medical research data is essential, regardless of being publicly- or industry-funded [25]. A new generation of citizen scientists takes this advocacy to a new level, increasing the need for open access in order to effectively implement studies and drive innovation [17]. For example, high school student scientist and advocate for open access Jack Andraka made use of what few non-paywalled articles he could find on the internet to invent a scholarship award-winning early detection test for pancreatic cancer [26], demonstrating the important advancements that can be made through increased public access to medical research. Table 1 outlines an additional example from the perspective of a member of our authorship group based at an LGBT community organization in Guangdong, China.

**Table 1:** A case study describing how open access medical publishing impacted the work of a non-governmental community-based LGBT organization in Guangdong, China

An Advocate’s Perspective on Open Access: Experiences from Zhitong Guangzhou LGBT Center Our community-based organization collaborated with a research team investigating the use of crowdsourcing to expand HIV testing among men who have sex with men in China. Throughout this collaboration, our organization was able to mobilize and engage a large number of community members to participate in the research. After the results of this study were published in an open access journal [27], we were able to use the freely-available article in an evidence-based report to open a dialog about new HIV testing resources and strategies with officials in the local Center for Disease Control and Prevention. This resulted in new funding and resources for our organization, which enabled us to implement the innovative findings of the study of community members’ ideas to promote HIV testing. It also has deepened our relationship with and trust between our organization and the government. With increased open access to medical research findings, more of these kinds of opportunities and cooperative efforts will be possible. The impact of open access is especially important in regions with limited opportunities to advocate for certain sensitive issues like expanded HIV services. Open access can help community-based organizations to access medical evidence to amplify their voice in a legitimate way and achieve their agendas for serving the local community. Additionally, open access publishing provides greater opportunities for inspiring people from the patient and/or affected community to become engaged in research and help to find solutions to health problems. Open access is important for advocates’ efforts to increase opportunities for community members to contribute their perspectives and wisdom on health issues, to corroborate (or challenge) medical findings, and to be a part of the research about them in a more intimate and personal way. Based on the experiences of our organization, open access is a revolution and represents the future for how to conduct and disseminate medical research.	

### Obligations to research participants

Research participants generously give their time and energy to research studies with the assumption that they are contributing to generalizable knowledge for the greater good [28]. Limiting access to research results limits the social value of research, which is an essential part of the ethical justification of doing research in the first place [29]. The assumption that the research has social value is reinforced by informed consent documents for research involving human subjects, which typically state that findings (positive or negative) will be broadly disseminated. When dissemination is conditional upon having access to paywall-protected research results, the social value of research is impeded, and the process of informed consent is compromised. Additionally, paywalls prevent research participants from accessing information about clinical trial results that their own time and efforts made possible, potentially undermining trust in medical research and impacting willingness to participate in future studies. Ultimately, paywalls hinder the ability of patient participants to genuinely act as co-creators of public knowledge.

### The public as patients

Finally, members of the public have an interest in expanding open access to medical research in their roles as patients. Members of the public express a strong preference for accessing health information through the internet [30], and among internet users, conducting an initial search for health information online has become a routine first step in the pathway to accessing health care [31]. Improving access to primary sources of medical literature through open access publishing could thus contribute to patient empowerment and the ability for patients to avoid misinformation that could impact their health and wellbeing, which in turn has implications for public health promotion at the population level. Patient advocacy groups are already making use of open access to help patients stay up-to-date on the latest medical advancements pertaining to specific conditions. For example, open access to medical information is a key strategy in the work of Melanoma Patient Network Europe: a network of melanoma patients, their caregivers, and advocates with the mission to provide evidence-based education about melanoma so that patients can be actively involved in their care [32]. Open access may be particularly important for those patients seeking information on complex or uncommon health conditions for which little information exists outside of academic publications. For example, individuals with rare diseases or genetic disorders (or the lay caregivers/advocates thereof) would be able to access the latest research about their health conditions without being blocked by a paywall [33]. For this reason, representatives of patient advocacy groups such as the M-CM Network – a research and advocacy organization for the rare genetic condition macrocephaly-capillary malformation syndrome – have called for greater open access to medical research as well as further engagement of patients in the open access movement [34].

### Caveats and counterpoints to open access for the public

We recognize that there are some important tensions in the argument for increased open access to medical research from the perspective of public stakeholders, as well as objections to open access that require careful consideration. The foremost tension to address is that open access should not be oversold as a total panacea for the involvement of public voices in medical research. Widespread open access to medical journals will not automatically democratize science, as there are many barriers and inequalities likely to persist in public information access beyond overcoming paywalls; for example, the dominance of English language publications and entrenched power structures in the global north [35]. Broader, systemic changes in resource distribution will be needed alongside increased open access in order to address these issues from the perspective of the global public and in the interest of global public health. Greater efforts to advance researcher-community collaboration, such as those experienced by a member of our authorship group (see Table 1), are also needed for successful public advocacy and innovation, which cannot be ensured through expanded open access alone.

We also recognize that the ideal of universal open access has a number of logistical challenges to contend with apart from the inclusion of a public rationale, with questions raised about who will pay for open access publishing, under what business model, and at what price. As a recent editorial in the *New England Journal of Medicine* has argued, there are necessary and unavoidable costs involved in academic publishing, including the expense of editorial processing and production staffing [36]. However, while the reality is that someone must pay the costs of publishing, there is arguably a substantial difference between ensuring a sustainable income for a journal to cover production costs, compared to publishers’ profit-driven annual subscription mark-ups to boost their bottom line. Publishers also increasingly profit from paywalls not only by raising subscription fees, but by actively reducing their production costs; for example, the move to online-only publishing has substantially decreased expenses associated with print publishing, resulting in year-over-year profit margin growth for shareholders [37]. From the perspective of public laypersons, the ‘need’ to ensure that corporate shareholders get paid as much return on investment as possible is not a particularly compelling counterpoint to universal open access to medical research.

There are also concerns with the way that for-profit journals benefit from offering an open access publishing option. In current open access publishing models, many of the costs associated with production are passed on to individual researchers who agree to pay a fee should their submission be accepted by the journal. This model has been criticized for the potential to create a two-tiered system in which peer review is not the sole deciding factor in whose research gets published, but additionally who can afford the fee [38]. This raises questions about research quality (a concern of relevance to the scientific community and general public alike) as well as entrenches the dominance of scientific outputs from richer countries. One potential way to address this concern is by shifting the burden of open access fee payment from individual researchers to research funders. This is one of the principles underlying Plan S: a strategy developed by a coalition of national research funders and charitable organizations (with support from the European Commission and the European Research Council) to ensure that the results of all research supported by grants from the coalition’s participating organizations are published only in open access journals, starting in 2021 [39]. Plan S would require participating research funders to cover open access publishing fees, eliminating not only embargo periods on scientific evidence, but also the financial disincentive for individual researchers to publish in open access journals [21]. Funders under Plan S will also pledge to monitor the transparency of journal publishing fees and potentially standardize fee funding in response to price fluctuations, as well as base research funding decisions on the assessment of the merits of the research rather than the prestige of the journals in which the results are published [40]. Collectively, the principles of Plan S operate on the underlying assumption that unrestricted, universal access to scientific knowledge through open access is a public good [41].

However, it should be noted that shifting the costs of open access to funders may also have unintended consequences. For example, independent researchers (including members of the public who are not affiliated with an academic institution) and graduate students without funding sources would not be able to have publishing costs covered by such strategies. Additionally, given vast inequalities in the amount of funding by discipline (e.g. that between the natural vs. social sciences) [42], requirements for funders to cover open access publishing costs may further exacerbate disciplinary gaps in research resources – subsequently resulting in systemic biases in the kinds of knowledge freely available for public access. Additionally, paying open access fees may be a financial strain for funders who inherently have an interest in keeping research costs low [43], potentially resulting in restrictions on research funds which would ultimately impact patients and the public at large. Thus while strategies like Plan S may be a step towards advancing open access as a public good, there are emergent questions about the potential inequalities and drawbacks of this model that will require consideration. Plan S continues to undergo revisions to both its workplan and implementation guidelines [44].

An additional caveat to the argument in favor of increased open access is that public *accessibility* of medical research requires more than simply making research freely available. Many journals are moving to completely digital formats, such that even in the absence of paywall barriers, those without ready computer/internet access will be excluded. Additionally, some measure of scientific literacy is necessary in order for laypersons to truly benefit from open access to medical research. Merely removing paywalls does not simultaneously address gaps in the comprehensiveness or navigability of research among the general public; if scientific literacy is not also addressed alongside open access, there is a risk that laypersons will draw inaccurate conclusions from articles they do understand. Increased open access may pose additional risks to the public rather than benefits, such as in the case of fraudulent research results [45] or materials published in predatory journals without sufficient peer review [46]. This may be of particular concern given that the availability of medical knowledge online has been shown to influence health-seeking behavior [31]. There has also long been concern with mass media sensationalization or distortion of research findings [47], which could potentially be exacerbated by increased open access to medical research. However, limiting access to medical research on the basis of risk management would be paternalistic at best; and at worst, it could be considered a violation of the human right of access to knowledge [48]. Rather than limiting access to research, we would argue the focus should instead be on increasing science literacy and improving the quality of academic publishing for the benefit of all; for example, by working to enhance researchers’ ability to identify predatory journals [49] and by tracking and reporting on retracted publications [50]. Greater efforts can also be made on the part of scientific writers to make research more linguistically and conceptually accessible [51]. Lay summaries accompanying publications may help to make open access research more navigable by members of the general public [52]. Recognizing the importance of lay summaries for public transparency and communication of clinical trial results, the European Medicines Agency has made the provision of lay summaries a requirement through the European Union Clinical Trials Regulation (EU CTR) 536/2014 [53]. This regulation will require a lay summary for all registered European Union clinical trials. Additionally, the inclusion of visual infographics summarizing key findings can help make research articles more readily shareable not only by academic peers but also members of the public press [54]. Further research is also needed to better understand how members of the general public find, use and share open access information so that efforts to improve navigability can be most effectively implemented [5, 31]. For example, one study of laypersons’ perspectives on open access to medical research found that patient participants desired not only increased open access and lay summaries as a complement to full-text articles, but also improvements to the discoverability of open access resources [52].

## Conclusions

Re-focusing the open access debate to include a broad range of public voices and an emphasis on public benefit is important. An academic publishing ecosystem that allows research outputs to be hidden behind paywalls – or that only makes knowledge available based on the ability of researchers to pay publishing fees – conflicts with the societal values of accountability, transparency, and scientific knowledge as a common good [55]. As the work of pushing forward the frontier of open access continues, there is more that can be done to serve public interests in terms of accountability, advocacy, research participation, and patient care. There are several practical strategies that can be implemented to this end. First, we call on public institutions to announce what percent of their library budgets go towards journal access subscriptions, fostering greater transparency. More robust advocacy for open access is also needed, both within and outside of academia. Research scientists should reflect on how their contributions to paywalled journals inadvertently hide results from study participants and the wider public. We encourage research participants to ask about how the results of their study will be disseminated, and specifically inquire about research groups’ intentions to publish in open-access journals. Finally, we call for better inclusion of public voices in the conversation, as it is the public whose paychecks underwrite government-sponsored research, whose advocacy changes the arc of the possible, and whose participation allows medical research to happen.

## Data Availability

Not applicable.
